# Doxycycline Hyclate Modulates Antioxidant Defenses, Matrix Metalloproteinases, and COX-2 Activity Accelerating Skin Wound Healing by Secondary Intention in Rats

**DOI:** 10.1155/2021/4681041

**Published:** 2021-04-17

**Authors:** Luciana S. Altoé, Raul S. Alves, Lyvia L. Miranda, Mariáurea M. Sarandy, Daniel S. S. Bastos, Elda Gonçalves-Santos, Rômulo D. Novaes, Reggiani V. Gonçalves

**Affiliations:** ^1^Departament of General Biology, Federal University of Viçosa, Viçosa, Minas Gerais 36570-900, Brazil; ^2^Departament of Structural Biology, Federal University of Alfenas, Alfenas, Minas Gerais 37130-001, Brazil; ^3^Departament of Animal Biology, Federal University of Viçosa, Viçosa, Minas Gerais 36570-900, Brazil

## Abstract

The main objective of this study was to investigate the action of doxycycline hyclate (Dx) in the skin wound healing process in Wistar rats. We investigated the effect of Dx on inflammatory cell recruitment and production of inflammatory mediators via *in vitro* and *in vivo* analysis. In addition, we analyzed neovascularization, extracellular matrix deposition, and antioxidant potential of Dx on cutaneous repair in Wistar rats. Male animals (*n* = 15) were divided into three groups with five animals each (protocol: 72/2017), and three skin wounds (12 mm diameter) were created on the back of the animals. The groups were as follows: C, received distilled water (control); Dx1, doxycycline hyclate (10 mg/kg/day); and Dx2, doxycycline hyclate (30 mg/kg/day). The applications were carried out daily for up to 21 days, and tissues from different wounds were removed every 7 days. Our *in vitro* analysis demonstrated that Dx led to macrophage proliferation and increased N-acetyl-*β*-D-glucosaminidase (NAG) production, besides decreased cyclooxygenase-2 (COX-2), prostaglandin E2 (PGE2), and metalloproteinases (MMP), which indicates that macrophage activation and COX-2 inhibition are possibly regulated by independent mechanisms. *In vivo*, our findings presented increased cellularity, blood vessels, and the number of mast cells. However, downregulation was observed in the COX-2 and PGE2 expression, which was limited to epidermal cells. Our results also showed that the downregulation of this pathway benefits the oxidative balance by reducing protein carbonyls, malondialdehyde, nitric oxide, and hydrogen peroxide (H_2_O_2_). In addition, there was an increase in the antioxidant enzymes (catalase and superoxide dismutase) after Dx exposure, which demonstrates its antioxidant potential. Finally, Dx increased the number of types I collagen and elastic fibers and reduced the levels of MMP, thus accelerating the closure of skin wounds. Our findings indicated that both doses of Dx can modulate the skin repair process, but the best effects were observed after exposure to the highest dose.

## 1. Introduction

The skin is a complex organ that serves as a barrier to protect the body from the external environment [1]. However, different aggressive agents, such as trauma and microorganisms, can affect the structure and functions of this organ. In the case of a lesion, there is an exposure of subcutaneous tissue, which provides a humid and nutritious environment for microbial proliferation and colonization [2]. An infected cutaneous wound increases the risks of chronification, reduces the quality of life, and causes a high mortality rate of patients [3]. Skin wounds represent a serious health problem worldwide and are frequently associated with high costs and inefficient treatments with limited efficiency [4]. The therapies available today are aimed at improving the healing of wounds by promoting their rapid closure. However, the control of infections is generally neglected [5]. As a result, it is desirable to develop therapeutic interventions that control the infection and increase cutaneous repair.

The repairing of cutaneous wounds is a process that involves a complex interaction between cells, extracellular matrix, blood vessels, and tissue growth factors. Furthermore, the process is separated into the phases of inflammation, proliferation (granulation), and tissue remodeling [6]. During the inflammation phase, there is a migration of leucocytes to the injured site, with the release of cell mediators. During the proliferative phase, there is a multiplication of keratinocytes, fibroblasts, and endothelial cells, resulting in the formation of granulation tissue, which is also rich in vessels and collagen type III [7]. The next phase is characterized by tissue remodeling and maturation, in which collagen III is replaced by collagen I, thus making the scar stronger and more resistant to mechanical forces [8, 9].

The skin healing process is known as acute or chronic, according to its duration and nature [7]. During a chronification process, there is persistent activation of COX way and neutrophils and macrophages release cytokines and chemokines, which attract more cells to the location of the inflammation and promote oxidative stress in the repairing tissue [10, 11]. The excess of proinflammatory mediators increases the peroxide of hydrogen (H_2_O_2_) and oxide nitric content, which accelerate the peroxidation of lipids and proteins [12]. The prooxidant mediators cause damage to the cutaneous tissue and delay the wound healing process. Therefore, a controlled inflammation process is necessary to avoid persistent tissue damage through the continued action of free radicals and reactive oxygen species (ROS) [13]. Associated with this, we can highlight that the skin healing environment is usually prooxidant and generally presents decreased synthesis and expression of the antioxidant enzymes, such as superoxide, glutathione, and catalase, which impair the healing environment [14].

In general, a desirable repair process results from a balanced process of synthesis and degradation of inflammatory mediators and pro- and antioxidant compounds consequently in the extracellular matrix components, especially collagen [15]. Matrix metalloproteinases (MMP) play an essential role in the turnover of the extracellular matrix (ECM) remodeling, by degrading collagen and noncollagenous elements, such as glycosaminoglycans, proteoglycans, cytokines, growth factors, and their receptors [16]. However, the overexpression of MMP may lead to uncontrolled degradation of ECM and delayed the wound healing process [17–19]. Thus, MMP modulatory drugs may cause an important impact on cutaneous tissue repair, with a particular effect on tissue inflammation and maturation. In this context, doxycycline (Dx) has already been described as an inhibitor of MMP activity [20], and its usage has already been proven in the modulation of tissue levels of collagen in cutaneous repair [21], which stimulates collagen deposition. In addition, Dx proved to be an important tool to inhibit the release of proinflammatory cytokines, such as tumor necrosis factor-alpha (TNF-*α*), IL-6, and IL-8 [22]. Thus, we believed that Dx may affect the cutaneous healing process. Although Dx is effective in treating various disorders [23–25], little is known about the role of Dx in skin tissue repair. Therefore, this study evaluated the effect of Dx on the viability of macrophages, monitored the inflammatory changes *in vitro*, and used an experimental model to understand the effect of Dx on inflammation, oxidative status, angiogenesis, and fibrogenic responses during wound healing in rats.

## 2. Materials and Methods

### 2.1. *In Vitro* Assays

#### 2.1.1. Cell Viability

Cell viability for RAW264.7 macrophages was evaluated by 3-[4,5-dimethylthiazol-2yl]-2,5-diphenyl tetrazolium bromide (MTT) assay as previously described [26, 27]. Cells were cultured in DMEM supplemented with 10% fetal bovine serum and 100 U/mL of penicillin/streptomycin in a humidified 5% CO_2_ 37°C incubator. To evaluate the effect of Dx on cell viability, RAW264.7 macrophages were seeded into 96-well plates at a density of 1 × 10^5^ cells/well in 200 *μ*L medium. After 24 h, the various concentrations of Dx (10, 30, 100, and 300 *μ*g/mL) were added to media, and the incubation continued for the next 24 h at 37°C and 5% CO_2_. The control (100% of growth) was carried out with cells cultured in medium only. The MTT solution was added to each well, and the cells were further incubated for 2 h, at 37°C. The MTT formazan generated during incubation was dissolved in DMSO, and the absorbance was measured at 570 nm. For each sample, the result was expressed as the percentage absorbance in relation to the control group.

#### 2.1.2. Macrophage Challenge with LPS

RAW264.7 macrophages cultured under the same conditions were seeded in 24-well polystyrene plates, at 2.5 × 10^5^ cells, and 1 mL of culture medium per well. After 24 h, the culture medium was replaced, and the RAW264.7 cells were incubated for 24 h with a fresh medium containing 10% FBS, without or with 100 ng/mL LPS (Sigma-Aldrich, St. Louis, Missouri, USA) and different Dx concentrations (10, 30, 100, and 300 *μ*g/mL). The control cells were treated with a fresh culture medium. After 24 h of incubation, the macrophages were harvested; the cell number was quantified and adjusted by cell counting in a Neubauer chamber. The cells were lysed with 100 mM NaCl/50 mM Tris-HCl buffer and centrifuged (1000*g* for 15 min at 4°C). The culture supernatant was collected to measure the prostaglandin production enzymatic analysis of MMP, cyclooxygenase-2, and N-acetylglucosaminidase.

*(1) Metalloprotease Activity*. The enzymatic activity of matrix metalloproteases in macrophage homogenate was measured using a fluorometric enzymatic kit, according to the manufacturer's instructions (ABCAM, Cambridge, MA, USA). The MMP activity was measured at 490 nm/525 nm (excitation/emission), as previously reported [28].

*(2) Cyclooxygenase-2 Activity*. An aliquot (100 *μ*L) of the macrophage homogenate was applied to measure the cyclooxygenase-2 (COX-2) activity, which was analyzed using a biochemical colorimetric kit, following the manufacturer's instructions (Cayman Chemical, Ann Arbor, MI, USA). The enzymatic assay was based on the peroxidase component of cyclooxygenases, in which the peroxidase activity was spectrophotometrically measured by monitoring the production of oxidized N,N,N′,N′-tetramethyl-p-phenylenediamine, at 590 nm.

*(3) Prostaglandin Production*. The prostaglandin E2 (PGE2) levels in the macrophage homogenate were quantified by the specific enzyme-linked immunosorbent assay (ELISA) kit, according to the manufacturer's instructions (Cayman Chemical, Ann Arbor, MI, USA). Briefly, 10 *μ*L homogenates were added to 96-well microplates previously sensitized with specific antibodies against PGE2. Prostaglandin levels were determined by spectrophotometry at 412 nm [29].

*(4) N-Acetylglucosaminidase Activity*. The activation of the RAW264.7 cells was measured based on the quantification of N-acetyl-*β*-D-glucosaminidase (NAG) activity, which is a lysosomal enzyme intensely produced by activated monocytes/macrophages [30]. The N-acetyl-*β*-D-glucosaminidase activity was measured in skin homogenate by using a commercial biochemical colorimetric kit, according to the manufacturer's instructions (Abcam, Cambridge, UK). This assay uses a synthetic p-nitrophenol derivative (R-*p*NP) as a NAG substrate and releases *p*NP, which is measured by spectrophotometry, at 400 nm.

### 2.2. *In Vivo* Assays

#### 2.2.1. Animals and Ethics

Fifteen healthy three-month-old male Wistar rats (*Rattus norvegicus*) (339.16 ± 16.25 g) were obtained from the Central House of the health and Biological Sciences Center, Federal University of Viçosa. These animals were randomly allocated in individual cages, which were cleaned daily and maintained under controlled environmental conditions (temperature: 22 ± 2°C, humidity: 60–70%, and light/dark cycle: 12/12 h). Commercial food and water were provided *ad libitum*. All the experiments were approved by the Animal Ethics Committee of the Federal University of Viçosa (registration no. 72/2017).

#### 2.2.2. The Procedure of Surgical Wounds

**Figure 1:**
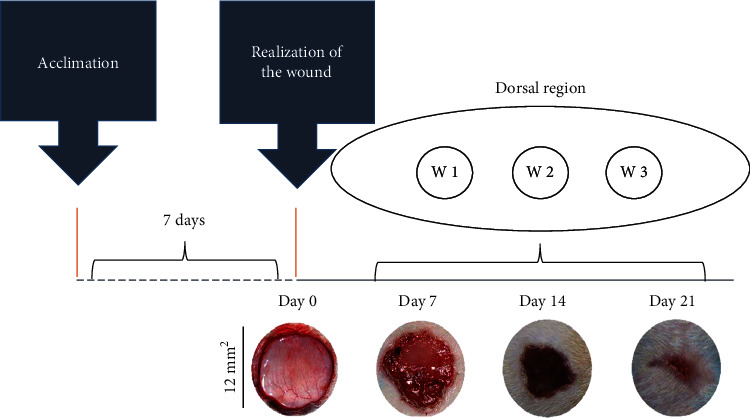
Representation of the experimental model of wound healing by secondary intention and time-dependent evolution of wound closure. The top image shows the distribution of the three excisional wounds in the back of the animal. The general appearance of wound closure from the initial wound (day 0) is represented by photographs. W1 (day 7), W2 (day 14), and W3 (day 21); macroscopic aspect of the wounds observed every 7 days. The wound areas were calculated on days 0, 7, 14, and 21 (mean ± SD), based on the digitized images.

The rats were anesthetized with an intraperitoneal injection of sodium pentobarbital (70 mg/kg). After anesthesia, the dorsolateral shaving of the animals was performed, and the area was cleaned with 70% alcohol. Three circular skin wounds of 12 mm diameter were created in the dorsolateral region of each rat by secondary intention, with surgical excision of the skin and subcutaneous cellular tissue using surgical scissors. The area of the wound was marked with violet crystal and measured with an analog caliper (Mitutoyo Sul Americana Ltda®, São Paulo, Brazil) [31]. No analgesia was administered after the surgical procedure since the application of drugs can alter cell migration and proliferation and compromise the skin repairing process. Tissue samples were obtained from different wounds at 7, 14, and 21 days, for histological and biochemical analyses, as presented in Figure 1.

#### 2.2.3. Experimental Design

The animals were randomized into three groups, with five animals in each group: C (distilled water, control); Dx1 (doxycycline 10 mg/kg/day), and Dx2 (doxycycline 30 mg/kg/day). The treatments were administered by gavage for 21 days. After this period, the animals were euthanized by cardiac puncture and exsanguination, after an anesthetic procedure. The doses were provided according to studies that used oral Dx for corneal reepithelialization in the rabbit model [32], and Dx was given by gavage to inhibit MMP-mediated vascular changes in hypertension in rats [33]. They found that many animals died after 100 mg/kg daily, thus suggesting that the therapeutic window for doxycycline may be rather narrow. Therefore, we decided to study the effects associated with the 10 and 30 mg/kg dose, because studies have not reported harmful effects on animals.

#### 2.2.4. Calculation of the Area and the Rate of Wound Contraction

The area and rate of contraction of the third wound were evaluated every 7 days, using images scanned with 320 × 240 pixels (24 bits/pixel) obtained by an Asus Zenfone 2 ZE551ML smartphone (ASUS, Taipei, Taiwan). The wound area was calculated by the formula *A* = *π* × (*r*)^2^, where *r* is the radius. The wound contraction index (WCI) was calculated by the ratio: initial wound area (*A*_o_) − the area on a given day (*A*_i_)/initial wound area (*A*_o_) × 100 [34, 35].

### 2.3. Histological and Stereological Analysis

The samples collected from the wounds, with tissue from the center of the lesion, were immersed in histological fixative for 24 h, dehydrated in ethanol, diaphanized in xylene, and immersed in paraffin. Histological sections (4 *μ*m thick) were obtained on a rotary microtome (Leica Multicut 2045, Reichert-Jung Products, Germany). We used 1 of every 20 sections to avoid repeating the analysis of the same histological area. The sections were stained with hematoxylin and eosin (HE) for the analysis of the fibroblasts and blood vessels [31]. Furthermore, the samples were stained with Sirius red for the analysis of collagen fiber types I and III [36]. Toluidine blue was used to identify mast cells [37], and Verhoeff was used to differentiate elastic fibers [14]. The slides were visualized and captured in a BX601 light microscope (Olympus, São Paulo, Brazil) coupled with a QColor-31 digital camera (Olympus, São Paulo, Brazil). Five images were selected at random using a 20x objective lens. For this analysis, a grid containing 256 points within a standard test area (AT) of 73 × 10^3^ *μ*m^2^ was superimposed over each image. The stereological parameters of volumetric density (*V*_v_) were calculated by counting the points that occurred over cells, blood vessels, types I and III collagen, and elastic fibers, using the ratio: *V*_v_ = PP/PT, where PP is the number of points occurring over the structures of interest and PT is the total number of points on the test system [31, 38]. Collagen fibers were analyzed according to the different properties of birefringence, as thick collagen fibers (type I) appear in shades of bright colors ranging from red to yellow, whereas thin reticular fibers (collagen type III) appear bright green under polarization [31, 39]. The mast cells were analyzed using a 40x objective lens. Ten microscopical fields were randomly analyzed in each histological section to obtain a total area (TA) of 1.96 mm^2^. The number of mast cells per unit of histological area was calculated according to the relation QA = *Σ* mast cells/TA [40].

### 2.4. COX-2 Immunohistochemistry

The histological sections (4 *μ*m thick) were dewaxed with xylene and hydrated in distilled water. Antigen recovery was performed with citrate buffer (pH 6) in a pressure cooker for 4 min. The sections were incubated for 10 min in 3% hydrogen peroxide to block endogenous peroxidase, followed by 15 min in 5% nonfat milk prepared at pH 7.6 TBST (1X Tris-buffered saline with 0.05% Tween 20). Then, the sections were incubated for 12 h, at 4°C, with a primary rabbit anti-COX-2 antibody (ab15191, Abcam, Cambridge, UK), at 1 : 1000 dilution. The slides were washed with TBST and incubated for 2 h at room temperature, with a ready-to-use secondary goat anti-rabbit IgG antibody conjugated with horseradish peroxidase (Dako EnVision™+ Dual Link System-HRP, Agilent, Santa Clara, CA, USA). The slides were washed with TBST, and the COX-2 marking was revealed with 0.5% 3,3′-diaminobenzidine for 5 min. Finally, the slides were released in ethanol, treated with xylene, and mounted with coverslips.

### 2.5. Biochemical Assays

Tissue fragments were collected from each wound, immediately frozen in liquid nitrogen (-196°C), and stored in a freezer at −80°C. The samples (200 mg) were homogenized in 2 mL phosphate-buffered saline (PBS) and centrifuged for 5 minutes at 10,000*g*, under refrigeration, at 5°C [14]. The supernatant and tissue pellets were separately collected and used in all biochemical analyses described below.

#### 2.5.1. Cyclooxygenase-2, N-Acetylglucosaminidase Activity, and Prostaglandin Production

Cyclooxygenase-2, N-acetylglucosaminidase activity, and prostaglandin E2 production in the scar tissue were measured with the aid of the same commercial kits used to quantify these parameters in the *in vitro* model. All measures were performed from intact skin (day 0) and scar tissue collected at days 7, 14, and 21 of wound healing. The enzymatic activity and prostaglandin levels were measured in tissue homogenate supernatant.

#### 2.5.2. Hydrogen Peroxide and Nitric Oxide Production

The hydrogen peroxide (H_2_O_2_) production was measured in supernatants of tissue homogenates. 50 *μ*L of supernatants were incubated with 50 *μ*L of *α-*Phenylenediamine dihydrochloride (OPD) and an equal volume of peroxidase type II 15 mmol/L. The conversion of absorbance into micromolar concentrations of H_2_O_2_ was calculated based on a standard curve, using a known concentration of H_2_O_2_. The results were expressed as *μ*mol/L [41].

Nitric oxide (NO) was indirectly quantified through the detection of nitrite/nitrate (NO_2_^−^/NO_3_^−^) levels by the standard Griess reaction [42]. 50 *μ*L of supernatants were incubated with an equal volume of Griess reagent (1% sulfanilamide, 0,1% N-(1-Naftil) ethylenediamine, and 2.5% H3PO4) and kept at room temperature for 10 minutes. The conversion of absorbance into micromolar concentrations of NO was obtained from a sodium nitrite standard curve (0–125 *μ*M) and expressed as NO concentrations (*μ*mol × L^−1^).

#### 2.5.3. Determination of Lipid and Protein Oxidation

Lipid peroxidation (LPO) was estimated according to the total malondialdehyde levels (MDA) [43]. The MDA concentration was determined by using the standard curve of known concentrations of 1, 1, 3, 3-tetramethoxypropane (TMPO). The results were expressed as *μ*mol × L^−1^ per mg of protein.

Protein oxidation was estimated from protein carbonyl content, which was measured using 2,4-dinitrophenylhydrazine (DNPH) [44], based on the carbonyl group reaction with DNPH. The pellets resulting from the tissue homogenates from previous extractions were used for quantification. The results were expressed as nmol per mL of protein.

#### 2.5.4. Superoxide Dismutase Activity

The activity of superoxide dismutase (SOD) was determined by the superoxide (O_2_^−^) and hydrogen peroxide reduction method, thereby decreasing the autooxidation of pyrogallol [45]. SOD activity was calculated as units per milligram of protein, with one unit (U) of SOD defined as the amount that inhibited the rate of pyrogallol autoxidation by 50%.

#### 2.5.5. Catalase Activity

The catalase (CAT) activity was evaluated according to the method described by Aebi [46], by measuring the rate of decomposition of hydrogen peroxide. One unit of CAT activity was calculated using the number of enzymes that decompose 1 mmol H_2_O_2_ for 1 min. The results were expressed as units of catalase/milligram of protein.

#### 2.5.6. Glutathione S-Transferase Activity

The glutathione S-transferase (GST) activity was measured using the method of Habig et al. [47]. Glutathione S-transferase activity was analyzed according to the formation of glutathione-conjugated 2,4-dinitrochlorobenzene (CDNB). One unit of GST activity was defined as the amount of enzyme that catalyzed the formation of one *μ*mol of product/min/mL. GST activity was expressed as U per milligram of protein.

#### 2.5.7. Evaluation of MMP-10 Cutaneous Activity

For the evaluation of MMP-10 activity, 200 mg samples of the skin were homogenized in 1 mL of 5 mM Tris-HCl (pH 7.4) buffer containing 0.15 M NaCl, 10 mM CaCl_2_, and 0.02% NaN_3_. After centrifugation at 10,000*g* for 30 min, the supernatant was collected for analysis of MMP activity. For such, an ELISA commercial immunoenzymatic kit was used according to the manufacturer's instructions (Sigma-Aldrich; Merck KGaA, Darmstadt, Germany).

### 2.6. Statistical Analysis

The statistical analysis was carried out using the GraphPad Prism 7 software system (GraphPad Software Inc., San Diego, Calif., USA). The results were expressed as mean and standard deviation (mean ± SD). The parametric data were compared using one-way ANOVA variance analysis, followed by the Student-Newman-Keuls *post hoc* test. The nonparametric data were compared using the Kruskal–Wallis test. Statistical significance was established at *p* ≤ 0.05.

## 3. Results

### 3.1. Impact of Dx on Macrophage Viability, Prostaglandin Production, and COX-2, MMP, and NAG Activity

**Figure 2:**
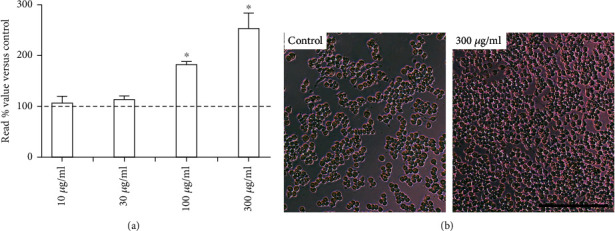
Effects of doxycycline hyclate (Dx) on cell viability. (a) RAW264.7 macrophages were treated with various doses of Dx (10, 30, 100, and 300 *μ*g/mL) for 24 h. (b) Representative photomicrographs showing cells in cultured medium (control group) and 300 *μ*g/mL Dx added to the medium (phase-contrast microscopy, bar = 200 *μ*m). The results are presented as the percentage absorbance of the control group. The data are expressed in the graphics as a mean and standard deviation. ^∗^Statistical difference compared to control cells (Student-Newman-Keuls test, *p* < 0.05).

The effect of Dx on macrophage viability is presented in Figure 2. No cytotoxicity was observed after exposing the macrophages to Dx. Furthermore, the highest cell proliferation was observed after macrophage incubation with 100 *μ*g/mL (182.03 ± 6.57) and 300 *μ*g/mL (253.05 ± 30.92) of Dx, which indicates a clear dose-dependent effect (Figures 2(a) and 2(b)).

**Figure 3:**
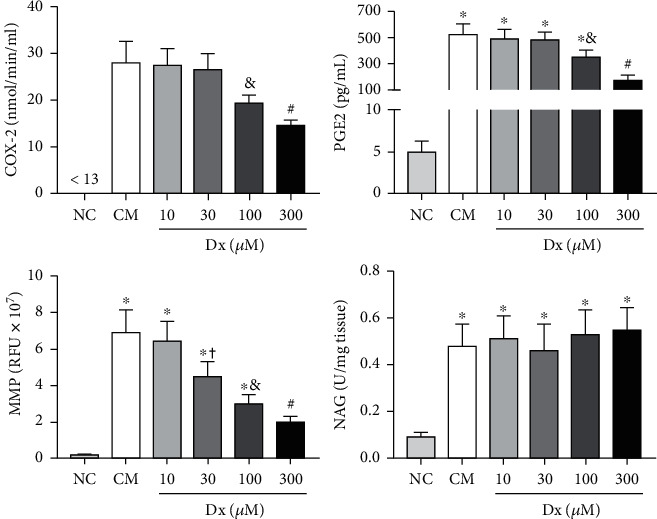
Effects of doxycycline hyclate (Dx) on cyclooxygenase-2 (COX-2), prostaglandin E2 production, and metalloproteinases (MMP) and N-acetyl-*β*-D-glucosaminidase (NAG) activity in LPS-stimulated RAW264.7 macrophages treated with various doses of Dx for 24 h. NC: not stimulated cells (not treated with LPS or Dx), CM: cells treated with culture medium containing LPS, and Dx: cells treated with culture medium containing LPS and Dx at 10, 30, 100, and 300 *μ*M. The data are expressed as mean and standard deviation. Statistical difference (Student-Newman-Keuls test, *p* < 0.05), compared to ^∗^NC, ^&^CM, Dx10, and Dx30, ^#^NC, CM, and Dx (10, 30, and 100), and + CM and Dx10.

In the *in vitro* analysis, Dx also reduced glycoprotein cyclooxygenase-2 (COX-2) in the Dx100 group when compared to CM, Dx10, and Dx30. The Dx300 group presented reduced COX2, in relation to NC, CM, Dx10, Dx30, and Dx100. The prostaglandin E2 levels were higher in CM and Dx (10, 30, and 100), when compared to the NC group. Dx100 presented a decrease in relation to CM, Dx10, and Dx30. Dx300 presented a decrease when compared to NC, CM, and Dx (10, 30, and 100). Metalloproteinases (MMP) presented a decrease in the CM, Dx10, Dx30, and Dx100 groups when compared to the NC group. The Dx100 group values were lower, compared to the CM, Dx10, and Dx30 groups. The Dx300 group presented lower values when compared to NC, CM, and Dx (10, 30, and 100). The NAG values were increased in all groups (CM, Dx10, Dx30, Dx100, and Dx300) when compared to the NC group (Figure 3).

### 3.2. Wound Area and Contraction Index

**Figure 4:**
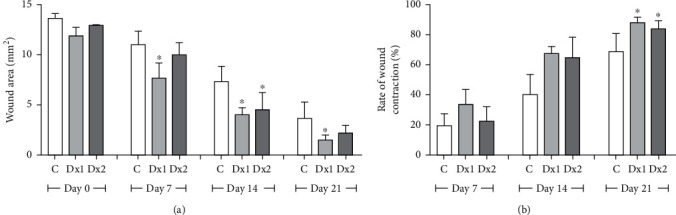
(a) Area (mm^2^) and (b) rate of wound contraction (%) in rats treated with doxycycline hyclate (Dx) after 7, 14, and 21 days of treatment. Dx1: doxycycline hyclate (10 mg/kg), Dx2: doxycycline hyclate (30 mg/kg). The data are expressed as mean and standard deviation. Statistical difference (Kruskal–Wallis test, *p* < 0.05) compared to the ^∗^control group.

The wound area was smaller on days 7, 14, and 21 in the Dx1 group, compared to the control animals, as well as on day 14, in the Dx2 group, in relation to the control group. The rate of wound contraction was higher in the Dx1 and Dx2 groups, compared to the control group, on day 21 (Figure 4).

### 3.3. Histopathological Results

**Figure 5:**
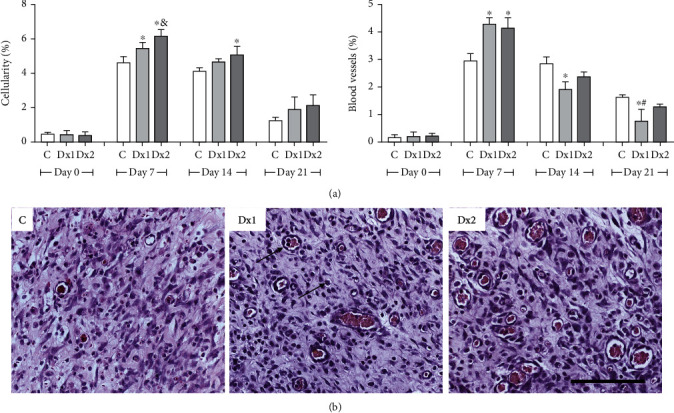
The proportion of cell nucleus and blood vessels (a) and representative photomicrographs showing cells and blood vessel distribution (b) in the scar tissue of rats untreated and treated with doxycycline hyclate (Dx), on day 7 (H&E staining, bar = 100 *μ*m). C: control, Dx1 = 10 mg/kg Dx, and Dx2 = 30 mg/kg Dx. In the graphics, the data are represented as mean and standard deviation. The statistical difference compared to the groups ^∗^C, ^&^Dx1, and ^#^Dx2 (Student-Newman-Keuls test, *p* < 0.05).

On day 7, the proportion of total cells in the tissue was higher in the Dx2 group, compared to other groups. On the same day, Dx1 showed a higher proportion of cells, compared to the control group. On day 14, Dx2 presented a higher proportion of cells in relation to the control group (Figure 5(a)). In relation to blood vessels, Dx1 and Dx2 groups showed an increased proportion of vessels when compared to the control group, on day 7. However, on day 14, the proportion of vessels was reduced in the Dx1 group, compared to the control animals. On day 21, Dx1 presented reduced blood vessels compared to the control and Dx2 groups (Figure 5(a)). The distribution of cells and blood vessels in the scar tissue of the different groups is shown in Figure 5(b).

**Figure 6:**
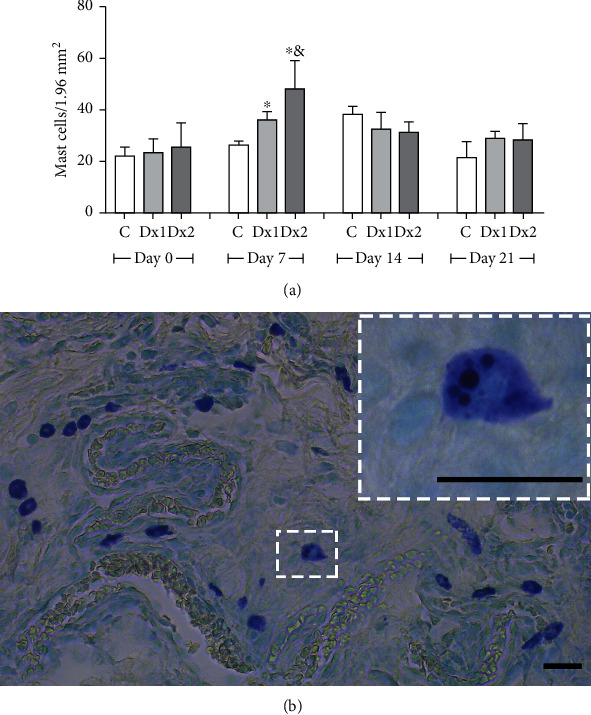
The number of mast cells (a) and representative photomicrograph showing mast cell distribution (b) in the scar tissue from a rat treated with doxycycline hyclate (Dx, group 2) on day seven of wound healing (Toluidine blue staining, bar = 50 *μ*m). C: control, Dx1 = 10 mg/kg Dx, and Dx2 = 30 mg/kg Dx. Data represented as mean and standard deviation. In the graphics, the data are represented as mean and standard deviation. The statistical difference compared to the groups ^∗^C and ^&^Dx1 (Student-Newman-Keuls test, *p* < 0.05).

The number of mast cells, on day 7, was higher in the Dx2 group, compared to the other groups. In addition, the Dx1 group presented a higher number of cells compared to the control group (Figure 6(a)). The distribution of mast cells in the scar tissue in the Dx2 group, on day 7, is shown in Figure 6(b).

**Figure 7:**
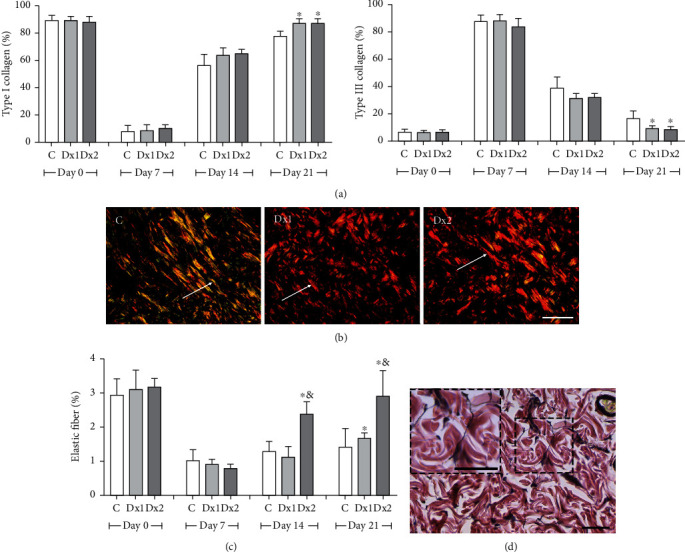
The proportion of type I and III collagen fibers (a), representative photomicrographs showing collagen fiber distribution, on day 21 ((b) Sirius red staining under polarized light, bar = 100 *μ*m), the proportion of elastic fibers (c), and representative photomicrograph showing elastic fiber distribution in group Dx2, on day 21 ((d) Verhoeff's elastic stain, bar = 50 *μ*m) in the scar tissue of rats untreated and treated with doxycycline hyclate (Dx). C: control, Dx = 10 mg/kg Dx, and Dx2 = 30 mg/kg Dx. In the graphics, the data are represented as mean and standard deviation. The statistical difference compared to the groups ^∗^C and ^&^Dx1 (Student-Newman-Keuls test, *p* < 0.05).

A higher proportion of type I collagen fibers were observed in the groups treated with Dx1 and Dx2, on day 21, when compared to the control group. The type III collagen fibers were reduced in Dx1 and Dx2, on day 21, in comparison to the control group (Figure 7(a)). The distribution of type I and type III fibers in the scar tissue of the different groups and the predominance of type I collagen fibers after treatment with Dx are shown in Figure 7(b). On days 14 and 21, the number of elastic fibers was higher in the Dx2 group, compared to the other groups (Figure 7(c)). The representative distribution of elastic fibers in the scar tissue in the Dx2 group, on day 21, is shown in Figure 7(d).

### 3.4. Biochemical Results

#### 3.4.1. Immunohistochemistry and COX-2, PGE2, and NAG Activity

**Figure 8:**
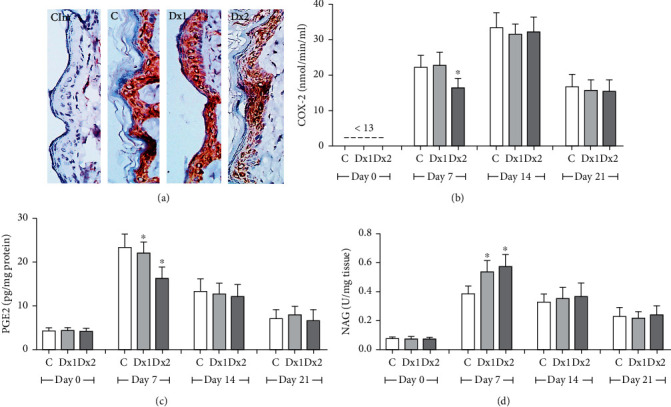
Immunohistochemical detection of cyclooxygenase-2 (COX-2) in the epithelial cells (a), COX-2 activity (b), prostaglandin E2 (PGE2) levels (c), and N-acetyl-*β*-D-glucosaminidase (NAG) activity (d) in the scar tissue from rats untreated and treated with doxycycline hyclate (Dx). Cim: control of the immunohistochemical method (the primary antibody was omitted in the technique), C: control, Dx = 10 mg/kg Dx, and Dx2 = 30 mg/kg Dx. In the graphics, data are represented as mean and standard deviation. ^∗^Statistical difference compared to group C (Student-Newman-Keuls test, *p* < 0.05).

Figures 8(a) and 8(b) show the results of the COX-2 analysis, which presented lower values in the Dx2 group when compared to control and Dx1, on day 7. These results were confirmed by the analysis of the photomicrographs, showing decreased COX-2 expression in Dx2, mainly in epithelial cells. In relation to PGE2, the Dx2 group showed lower values compared to the other groups, on day 7 (Figure 8(c)). The macrophage accumulation/activation was proven by increased NAG values in the Dx1 and Dx2 groups when compared to the control (Figure 8(d)).

#### 3.4.2. Oxidative Stress Markers

**Figure 9:**
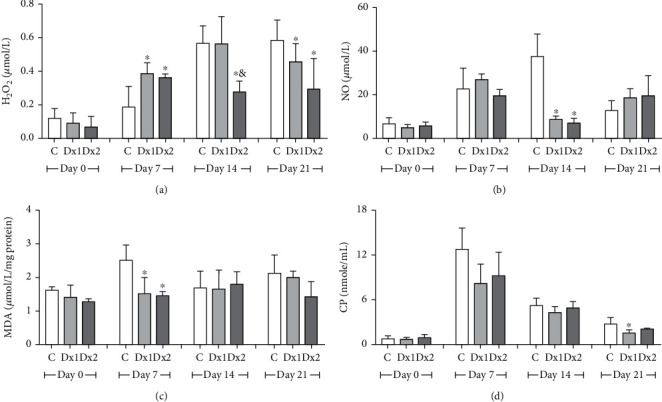
Levels of oxidative stress markers in the tissue: (a) hydrogen peroxide (H_2_O_2_), (b) nitrite and nitrate (NO_2_^−^/NO_3_^−^), (c) malondialdehyde (MDA), and (d) carbonyl proteins (CP) in the scar tissue of rats untreated and treated with doxycycline hyclate (Dx). C: control, Dx1 = 10 mg/kg Dx, and Dx2 = 30 mg/kg Dx. Data are represented as mean and standard deviation. The statistical difference compared to the group ^∗^C and ^&^Dx1 (Student-Newman-Keuls test, *p* < 0.05).

On day 7, H_2_O_2_ levels were higher in the Dx1 and Dx2 groups in relation to the control group. On day 14, Dx2 showed lower H_2_O_2_ levels in relation to the control and Dx1 groups. On day 21, Dx2 showed lower levels of H_2_O_2_, when compared to the control group (Figure 9(a)). Nitrite and nitrate levels were lower in the Dx1 and Dx2 groups, compared to the control group, on day 14 (Figure 9(b)). Concerning malondialdehyde, the levels were lower in the Dx1 and Dx2 groups, on day 7, when compared to the control (Figure 9(c)). On the other hand, reduced carbonyl protein levels were identified in the group Dx1, compared to the control group, on day 21 (Figure 9(d)).

#### 3.4.3. Antioxidant Enzymes and Metalloproteinase-10

**Figure 10:**
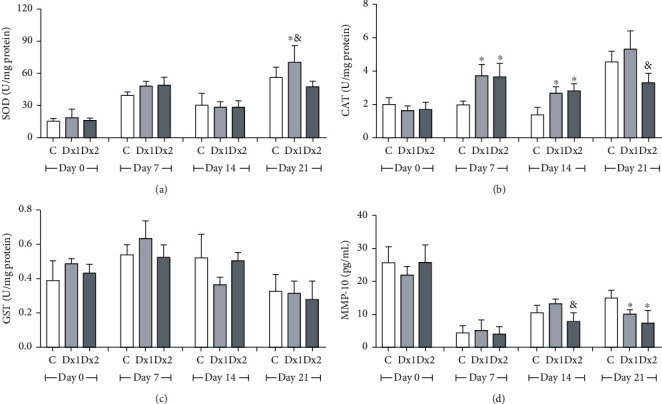
Levels of (a) superoxide dismutase (SOD), (b) catalase (CAT), (c) glutathione S-transferase (GST), and (d) metalloproteinase-10 (MMP-10) in the scar tissue of rats untreated and treated with doxycycline hyclate (Dx). C: control, Dx1 = 10 mg/kg Dx, and Dx2 = 30 mg/kg Dx. The data are represented as mean and standard deviation. The statistical difference compared to the group ^∗^C, ^&^Dx1, and Dx2 (Student-Newman-Keuls test, *p* < 0.05).

Superoxide dismutase activity was higher in the Dx1 group, on day 21, when compared to the Dx2 group (Figure 10(a)). Catalase activity was higher on days 7 and 14, in the Dx1 and Dx2 groups when compared to the control. On day 21, CAT activity was lower in Dx2, concerning the Dx1 group (Figure 10(b)). The glutathione S-transferase values were not significantly different over the trial period (Figure 10(c)). On day 14, MMP-10 levels were lower in the group Dx2 compared to Dx1. On day 21, both groups treated with Dx presented lower levels of MMP-10 when compared to the control group (Figure 10(d)).

## 4. Discussion

Doxycycline constitutes a large group of broad-spectrum antibiotics derived from tetracycline [48]. Studies have suggested that Dx presents therapeutic activities unrelated to its antimicrobial activity [49, 50]. The present study used an integrated cellular and tissue analysis to evaluate the effect Dx when administrated orally in Wistar rats, to repair their skin. Therefore, we observed that Dx was effective for complete wound closure and increased the rate of wound contraction. This effect is possibly associated with Dx antimicrobial and anti-inflammatory capacity [49, 51]. These results showed that Dx has beneficial action for the treatment of skin lesions. However, studies investigating the action of this drug on cutaneous repair are still scarce and limited, mainly when related to its antioxidant capacity. In a recent systematic review, we found positive results of antibiotic therapy for the treatment of cutaneous wounds. However, there is limited evidence that Dx can exert beneficial effects on the treatment of skin lesions *in vivo* [52]. In this review, sulfonamides were the antibiotic most commonly used, and doxycycline was tested in only one study [52]. As the main mechanisms involved in the cutaneous repair after Dx exposure remain unknown, studies using immunological, biochemical, and oxidative markers are required.

Due to its potent bacteriostatic properties, Dx is an effective antibiotic to treat diseases, such as syphilis [23], periodontal diseases [24], pneumonia [53], and cholera [54]. Although the effect of Dx on cutaneous repair is still poorly explored, this drug increases collagen deposition [55], stimulates tissue reepithelization [20] and favors the elimination of reactive oxygen species, thereby preventing or reducing pathological tissue destruction [56]. In addition, Dx changes the proliferation of inflammatory cells [57]. In this sense, we observed that Dx increased macrophages proliferation *in vitro*, exerting a potential dose-dependent immunoregulatory activity. This effect was aligned with increased macrophage activation, which was indicated by the upregulation of NAG activity induced by all doses of Dx. Conversely, Dx-treated macrophages exhibited a dose-dependent decrease in COX-2 and PGE2 levels, which indicates that macrophage activation and COX-2 inhibition are regulated by Dx through potentially independent mechanisms, which requires further investigation. However, there is evidence that different drugs with antimicrobial and anti-inflammatory properties such as nimesulide, ibuprofen, and amoxicillin can differentially regulate macrophages activity (i.e., endocytosis and nitric oxide production) and cytokine and prostaglandin secretion [58–60], which corroborates relative independence of these metabolic processes. Thus, this specific effect of Dx on COX-2 and macrophage activity is potentially relevant in wound healing, since it can modulate the intensity of the inflammatory response without inhibiting cell activation in response to antigenic stimulation, which exerts an essential protective role against infections.

In addition, our results also revealed an increased overall amount of scar tissue cells, highlighted by a high amount of mast cells. This was mainly observed when we used higher doses of Dx and in the early stages of the healing process. Mast cells are important for the inflammation phase of the repair process, as they promote the activation of macrophages and the formation of granulation tissue, cell migration, and maturation of collagen fibers [61]. Therefore, we believe that Dx can stimulate the process of cell migration and speed up the closure of the wound and consequently decrease the risk of infection. On the other hand, we observed that Dx stimulates an increase in the proportion of type I collagen, associated with a low proportion of type III collagen, after 21 days of treatment. These characteristics are very important since a high proportion of type I collagen fibers increases tissue resistance and force [62]. These collagen fibers exhibit covalent bonds and are more common in intact skin [63]. In general, at the start of the wound healing process, it is observed high deposition of type III collagen, but as the process progresses, the type III collagen fibers are replaced by type I collagen fibers [14], which makes the tissue more resistant to traction. About elastic fibers, after treatment with Dx, there was an increase in this fiber in the extracellular matrix in the scar tissue, especially in the final phase of tissue remodeling. Similarly, the role of Dx on elastic fibers was analyzed by Chung et al. [64], in the Marfan syndrome, in which Dx preserved the elastic fibers in the thoracic aorta, probably by inhibiting the action of metalloproteinases. Thus, our findings show the induction of the proliferative capacity of cells and, consequently, the synthesis of the extracellular matrix after the application of Dx.

Cellularity and neovascularization are essential to the process of wound healing, especially in the inflammatory phase [6]. A good vascularization can provide oxygen and nutrients for the cells, which are important for the recovery of injured tissue [65]. Besides, the formation of new vessels is directly linked to the formation of a new matrix, called granulation tissue, rich in vessels and cells. When the process advances, the number of vessels and cells decreases, but the number of fibers increases, which characterizes mature scar tissue [66]. Therefore, new effective treatments should promote intense neoangiogenesis at the start of the repair process and reduce the levels of vessels at the end of the process. For Altoé et al. [52], the tetracycline class represented by doxycycline increases collagen organization and, consequently, the rupture force of the wound. Corroborating the current evidence, our findings indicate that Dx stimulated the increase of new vessels at the start of the wound process (day 7) and the number of cells, possibly due to the intense migration and activation of the inflammatory cells proven by the increased NAG, at the same time. On the other hand, reduced vessels and cells were observed, while fibers increased in more advanced phases of the wound repair. These changes are very important for the evolution of wound healing and provide resistance and force for the new tissue.

Interestingly, Dx was also effective in attenuating COX-2 activity and PGE2 only in the initial stage of wound healing (day 7). As this inhibition occurred simultaneously to increased cellularity and NAG activity in the scar tissue, the main source of COX-2 activity and PGE2 seems to be unrelated to the increased recruitment of immune cells, including macrophages. Corroborating this proposition, we identified that animals treated with the higher dose of Dx presented a reduced COX-2 expression in the scar tissue, which was limited to epidermal cells. Thus, our findings indicated that keratinocytes are the primary targets of Dx-induced COX-2 downregulation, which represents an effect potentially limited to the initial stages of wound healing. From COX-2 activity, intense PG levels are detected after skin injuries, which are potent proinflammatory effectors that attract immune cells, stimulate fibroblast, and endothelial cell proliferation and metabolism in the wound area [11]. COX-2 played a central and regulatory role in the arachidonic acid pathway, by regulating hemostasis and inflammation [67] and directly impacting the development of the subsequent phases of wound healing [68]. Thus, controlling the inflammation from COX-2 downregulation may be a relevant strategy by which Dx accelerates the onset of the proliferative and remodeling phases, stimulating the resolution of the healing process. Since PG release mediated by COX-2 activation triggers prooxidant mechanisms, Dx potentially attenuates the oxidative stress as well, which can prolong the inflammatory phase due to secondary reactive molecular damage [49].

The production of intense free radicals is usually observed in the initial stages of the healing process. In skin lesions, phagocytic immune cells produce free radicals in a process known as respiratory burst [69]. Free radicals, reactive species of oxygen (ROS), and reactive species of nitrogen (RNS) promote cellular oxidative stress, damaging membranes, proteins, and genetic material [70, 71]. In respect to ROS, it is important to consider the presence of peroxide hydrogen (H_2_O_2_), known as an important marker of redox metabolism and more commonly found during the inflammatory phase of the healing process [72]. In our study, as expected, the levels of H_2_O_2_ increased on the 7th day in the groups treated with Dx, corresponding to the inflammatory phase, but decreased in the later phases, on days 14 and 21, in the Dx_2_ group. In this case, the excess of H_2_O_2_ probably occurred in the inflammatory phase, due to cell migration, of neutrophils and macrophages, which release mediators and reactive oxygen species during phagocytosis [73]. Also, we believe that, in this phase, the activation of nicotinamide adenine dinucleotide phosphate (NADPH) takes place, which generates H_2_O_2_ and activates cell proliferation and apoptosis. In our study, we believed that an increased number of macrophages, proven by *in vitro* and *in vivo* analysis of NAG, could justify the increased level of H_2_O_2_ in the early repair process. On the other hand, H_2_O_2_ and nitric oxide (NO) showed decreased levels during day 14, which indicates that Dx was efficient to inhibit lipid peroxidation and protein oxidation in the later phase of the process. However, the excess of NO may cause irreversible damage in the cells, impaired homeostasis, and the activation of various signaling cascades, such as mitogen-activated protein (MAP) or c-Jun N terminal kinase (JNK), thus causing cell degeneration and death [74]. In general, it is difficult to measure NO due to the short half-life in tissues. NO, nitrite (NO_2_), and nitrate (NO_3_) are the markers usually employed [75, 76]. As a result of this, in our study, we measured the content of nitrite and nitrate in the scar tissue of Wistar rats.

Markers of oxidative stress are important tools to evaluate the redox balance of the healthy and damaged tissues [77]. MDA is a compound of three carbons synthesized from the peroxidation of polyunsaturated fatty acids, generally formed during the oxidation of the cell membrane lipids [78]. During cutaneous repair, MDA usually increases, mainly in the inflammatory phase, which indicates damaged tissue and slow wound closure [79]. In our study, we observed reduced levels of MDA on day 7, after treatment with Dx1 and Dx2, which indicates that doxycycline can help to positively modulate the redox status of tissue in recovery. Similar results were found by Nogueira et al. [80], after the induction of convulsive lesions, following the application of pilocarpine, a parasympathomimetic alkaloid extracted from jaborandi leaves. These authors showed that Dx reduced lipid peroxidation in the brain, thus protecting the tissue from the action of free radicals. Another important marker of the action of ROS and RNS in the tissue is the high content of carbonylated protein, which indicates protein oxidation [66, 81]. In the present study, after treatment with Dx, only using the lower dose, reduced levels of protein carbonylation were observed, suggesting the antioxidant secondary action of this drug. Serra et al. [82] evaluated the antioxidant action of doxycycline and demonstrated that this drug, as well as other tetracyclines, is similar to vitamin E, mainly due to the presence of a phenolic ring with multiple substitutions. This phenolic ring reacts with the free radical and generates a phenolic radical, which is relatively stable and not reactive to cellular components [83]. In this sense, in addition to acting as an antimicrobial, Dx also presents antioxidant secondary action, which protects the recovering tissue.

To maintain redox homeostasis in an injured tissue the action of the antioxidant defense system is necessary, which exhibits components, such as superoxide dismutase (SOD), catalase (CAT), and glutathione S- transferase (GST) [84, 85]. SOD removes superoxide radical, which is highly dangerous and destructive for the cells [86]. CAT enzyme accelerates the passage of electron and reduces the residence of H_2_O_2_ inside the cells, consequently avoiding the harmful effect of this compound on the tissues [87]. This means that, for a drug to be considered effective to treat lesions caused by free radicals, it is highly desirable that it stimulates the transcription and translation of these antioxidant enzymes. Our findings demonstrated that Dx increases SOD and CAT activity in the tissue. Increased CAT was mainly found in the inflammatory phase, which corroborates our findings in relation to the levels of H_2_O_2_. We believed that increased SOD and CAT reduced superoxide ion (***O***_***2***_^−^) and H_2_O_2_, thus protecting the tissue.

The imbalance between collagen synthesis and degradation is a common feature in cutaneous lesions, mainly in infected wounds [88]. A common consequence of this imbalance is the formation of hypertrophic scars and keloid, which results in fibrosis and loss of tissue function [8]. MMPs are enzymes found in acute or chronic skin wounds, which regulate the deposition of collagen and degrade the extracellular matrix, which is essential for wound reepithelization [19]. The excess of these enzymes may cause disorganization in the epithelium, cell-cell contact changes, and increased apoptosis of fibroblasts and keratinocytes [89]. In our study, we observed decreased levels of *MMP* in *in vivo* analysis, after treatment with Dx1 and Dx2, which corroborates our findings for decreased wound area and increased rate of wound contraction in these groups. For the healing process to occur smoothly and efficiently, a balance between the synthesis and degradation of collagen is required. If there is a predominance of MMP in the tissue, an exacerbated degradation of the extracellular matrix may occur, which compromises wound closure [19]. Other studies demonstrate that the administration of Dx speeds up the closure of cutaneous wounds by inhibiting MMP-9 [82] and accelerates the recovery of gastric wounds by inhibiting MMP-2 and H_2_O_2_ [90]. The balance in the synthesis of this enzyme is required for efficient cutaneous repair and can facilitate cellular migration and accelerate the recovery of the tissue.

## 5. Conclusions

Taken together, our findings indicate that both doses of Dx can modulate the repair of skin wounds in rats. However, in general, the best results in cellularity, mast cells, elastic fibers, hydrogen peroxide levels, and metalloproteinase-10 were observed after exposure to the highest dose of doxycycline hyclate (30 mg/kg). *In vitro*, our findings showed that Dx increased macrophage proliferation but decreased the COX-2 and PGE-2 levels. This indicates that macrophage activation and COX-2 inhibition are possibly regulated by independent mechanisms. Our findings showed that, *in vitro*, despite the increased in the number of cells in the initial phase of wound healing, there was a reduction in the expression of COX-2, which was limited to epidermal cells. Therefore, our results indicated that keratinocytes are the primary targets of Dx-induced COX-2 downregulation, and this capacity of modulating the intensity of the inflammatory response without inhibiting cell activation can play an important role in the favorable evolution of the healing process. In addition, since the COX-2 is involved in prooxidant mechanisms, the regulation of this pathway by Dx also favored the oxidative balance into the cell and protected the molecules against the action of free radicals, showing an antioxidant potential in cutaneous wound repair. This information may be relevant for the selection of the treatment, mainly in the acute phase of cutaneous wound healing.

## Data Availability

The data used to support the findings of this study are available from the corresponding author upon request.
