# EXPRESSION OF CONCERN: Doxycycline Hyclate Modulates Antioxidant Defenses, Matrix Metalloproteinases, and COX‐2 Activity Accelerating Skin Wound Healing by Secondary Intention in Rats

**DOI:** 10.1155/omcl/9829603

**Published:** 2026-10-29

EXPRESSION OF CONCERN: S.L. Altoé, R.S. Alves, L.L. Miranda, et al., “Doxycycline Hyclate Modulates Antioxidant Defenses, Matrix Metalloproteinases, and COX‐2 Activity Accelerating Skin Wound Healing by Secondary Intention in Rats,” *Oxidative Medicine and Cellular Longevity*, 2021, 4681041, https://doi.org/10.1155/2021/4681041.

This Expression of Concern is for the above article, published online on 19 April 2021 in Wiley Online Library (http://wileyonlinelibrary.com), and has been issued by agreement between the Journal Editor‐in‐Chief, Jeannette Vasquez Vivar; and John Wiley & Sons Ltd. The Expression of Concern has been agreed due to concerns that have been reported on PubPeer [1] and to the publisher directly, regarding Figure 2b. Specifically, there are unexpected, repeated elements within the 300 µg/mL panel in Figure 2b.

After evaluation by state‐of‐the‐art image analysis tools and also by a cell biologist within the publisher, the existence of unexpected, repeated elements has been verified. The authors initially stated that the duplication was introduced during figure preparation but then later stated that there is no duplication and the image is correct.

Therefore, the journal has decided to issue an Expression of Concern to inform and alert readers.
